# *Gordonia* sternal wound infection treated with ceftaroline: case report and literature review

**DOI:** 10.1099/jmmcr.0.005113

**Published:** 2017-09-13

**Authors:** Kevan Akrami, Joelle Coletta, Sanjay Mehta, Joshua Fierer

**Affiliations:** ^1^​ Department of Medicine, Division of Infectious Disease, University of California, San Diego, 9500 Gilman Drive # 0711 La Jolla, CA 92093-0711, USA; ^2^​ Department of Cardiothoracic Surgery, Veterans Affairs, 3350 La Jolla Village Dr, San Diego, CA 92161, USA; ^3^​ Department of Cardiothoracic Surgery Sulpizio Cardiovascular Center, University of California, San Diego, 9434 Medical Center Drive La Jolla, CA 92037, USA; ^4^​ Department of Medicine, Division of Infectious Disease, Veterans Affairs, 3350 La Jolla Village Dr, San Diego, CA 92161, USA

**Keywords:** *Gordonia bronchialis*, sternal wound infection, ceftaroline, cardiac surgery

## Abstract

**Introduction.** Case reports have emerged with identification of *Gordonia bronchialis* infections including sternal wound infections and foreign bodies such as central lines and shunts.

**Case presentation.** We present a case that demonstrates the need to consider *Gordonia* infection as a cause of sternal wound infection and highlights the utility of novel diagnostics to aid in the identification of unusual pathogens that can cause post-operative infections. We report here the first successful use of ceftaroline for treatment of a *G. bronchialis* sternal wound infection.

**Conclusion.** There are only case reports and *in vitro* assays to date to guide treatment of this infection, and we now add ceftaroline as a new drug to consider, though adequate surgical debridement is paramount.

## Abbreviations

CABG, coronary artery bypass grafting; CT, computed tomography.

## Introduction

Sternal wound infections following coronary artery bypass grafting (CABG) are rare occurrences with incidence ranging from 1–4 % [1–3]. Variables reported to increase the risk of post-operative sternal wound infections include bilateral internal mammary artery grafting (presumably from decreased sternal blood flow), diabetes mellitus, chronic obstructive pulmonary disease, peri-operative haemodynamic instability including use of vasopressor medication, and obesity [4]. Measures to decrease surgical site infections following cardiac surgery have focused on peri-operative chlorhexidine washing and antibiotics, sternal closure techniques, glycemic control and implantable antibiotics amongst others [4]. *Gordonia* sternal wound infections were first described in a case series published in 1991 wherein a single circulating scrub nurse nasally colonized with *Gordonia bronchialis* was identified as the vector for contamination of the surgical wounds [5]. Subsequent case reports have emerged with identification of *G. bronchialis* infections with foreign bodies including central lines and shunts (Table 1).

**Table 1.** Case reports of surgical site infections due to *Gordonia bronchialis*

Type of infection (no. of cases)	Type of procedure	Year of publication (references)
Sternal osteomyelitis	Sternotomy	Current case
Sternal wound	Sternotomy	2016 [11]
Subcutaneous abscess	Needle injection	2016 [12]
Peritonitis (2)	Peritoneal dialysis	2015, 2014 [13, 14]
Sternal wound	Sternotomy	2014 [15]
Sternal osteomyelitis	Sternotomy	2014 [16]
Sternal osteomyelitis	Sternotomy	2013 [17]
Sternal wound (3)	Sternotomy	2012 [8]
Tibial osteomyelitis	Arthroscopy	2012 [18]
Sternal wound (7)	Sternotomy	1991 [5]

## Case report

**Fig. 1.**
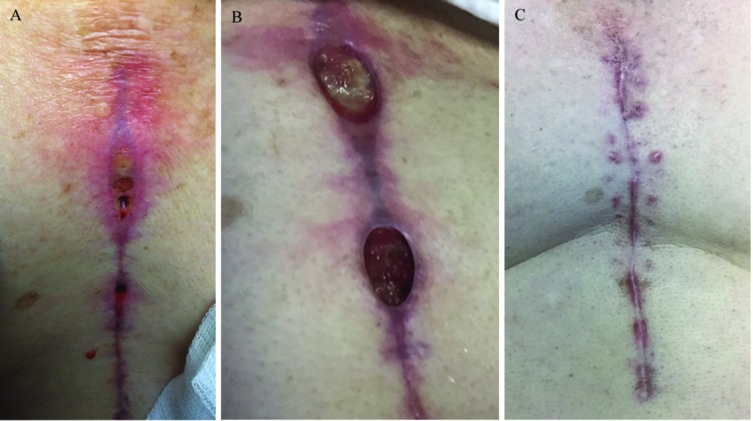
Images of the coronary artery bypass grafting wound from which *G. bronchialis* was isolated. (a) 8 weeks post-operative. (b) 12 weeks following initial surgery. 8 cm long sternal incisional wounds with surrounding erythema and clear drainage most prominent at cephalic location. (c) Complete resolution of incision 6 weeks post-debridement.

A 69-year-old man with well controlled diabetes mellitus underwent three vessel CABG at our hospital, including internal mammary and saphenous vein grafts performed using cardiopulmonary bypass. Six weeks post-operatively he presented to an outside hospital with erythema and drainage at the cranial end of the sternotomy incision (Fig. 1a). He was found to be afebrile and haemodynamically stable. Computed tomography (CT) imaging did not identify a drainable fluid collection. Cultures of the drainage reportedly grew scant diphtheroids. Despite empiric treatment with intravenous vancomycin for one month, he noted progressive enlargement of his wound with worsening drainage and chest pain. This prompted referral to our institution, 12 weeks after the bypass surgery. The patient did not have any systemic symptoms of illness. His physical exam was notable for being afebrile with normal vital signs, and having tenderness, erythema and drainage at the cephalic end of his sternal incision (Fig. 1b).

Further history obtained from the patient at that time revealed that he repairs industrial motorized fishing reels from around the United States. The rods often arrived covered in seaweed and barnacles. However, he did not handle the fishing gear himself. There were no other reported environmental exposures.

## Investigations

Laboratory results were notable for mild elevation in CRP (25.7 mg l^−1^, normal ≤8 mg l^−1^) with normal white blood cell count (7000 cells µl^−1^, normal range 4500–10000 cells µl^−1^). CT chest with IV contrast was notable for dehiscence of the manubrium and non-fused sternum with lytic and sclerotic changes concerning for sternal osteomyelitis. Given these findings, the patient underwent debridement of the sternal wound and wire removal. Pathology from the debridement demonstrated mostly granulation tissue.

## Diagnosis

**Fig. 2.**
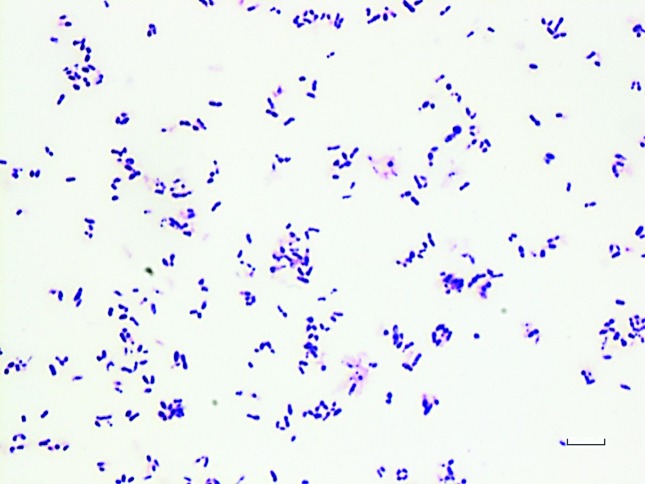
Gram stain made from a colony of *Gordonia bronchialis* isolated from a wound culture, demonstrating small pleomorphic Gram-positive rods. Bar, 10 µm.

Cultures from the operating room initially grew a few colonies of methicillin-resistant *Staphylococcus epidermidis* (MRSE) with a vancomycin MIC of 2 µg ml^−1^, but after 72 h there was robust growth of a Gram-positive rod (Fig. 2) that grew on blood, chocolate and Lowenstein–Jensen media, as well as in liquid mycobacterial media (VersaTrek; ThermoFisher). The organism was catalase-positive. Further testing using Vitek2 (bioMérieux) and by API strips (bioMérieux) led to low discrimination identification of either *Corynebacterium jeikeium* or *Microbacterium* spp.*, Gordonia, Dietzia* and *Nocardia*, respectively.

The isolate was sent out for further analysis by mass spectrometry using the MALDI Biotyper (Bruker) and returned with probable identification of *Gordonia bronchialis* (score 1.83). DNA extraction and PCR-amplification of the DNA gyrase region of *Gordonia* spp. with a set of novel PCR primers, followed by standard Sanger sequencing, confirmed the identification with 99 % similarity to previously reported *Gordonia bronchialis* sequences (GenBank accession number AB438182).

## Treatment

By disc diffusion testing, our *G. bronchialis* isolate appeared susceptible to penicillin, gentamicin, levofloxacin, minocycline, vancomycin, linezolid, tetracycline and erythromycin, although there are no established criteria for evaluating zone sizes for this organism. Given previous treatment with vancomycin without improvement, we looked for an alternative drug. Ceftaroline susceptibility was tested by Etest (bioMérieux) and the MIC was 0.19 µg ml^−1^, but there is not a Clinical and Laboratory Standards Institute (CLSI) breakpoint interpretation for this organism. We decided to treat this infection with ceftaroline at 600 mg every 8 h for 8 weeks, at which time the wound was closed (Fig. 1c).

## Outcome and follow-up

Eight months after treatment ended the wound was still closed, inflammatory markers normalized and the patient has remained pain-free.

## Discussion

Species of the genus *Gordonia* were previously included in the genus *Rhodococcus* but based on differences in 16S rRNA gene and gyrase subunit B sequences they were moved to a new genus. Species of the genus *Gordonia* are weakly acid-fast, aerobic, nocardioform actinomycetes that are found in soil, sewage and freshwater [6]. In the clinical microbiology laboratory they can be difficult to identify, and may be dismissed as skin or culture contaminants due to their diphtheroid-like appearance. Of note, in a large retrospective series *Corynebacterium* are rarely listed as a cause of sternal wound infections [7]. The case we present here appears to have been an isolated infection that presented several weeks following surgery. No subsequent cases have occurred in our hospital.

As with prior case reports (Table 1), in this case debridement was the primary modality of treatment with adjuvant antibiotics. *Gordonia* infections are exceedingly rare, primarily reported as surgical site infections, so there is little clinical experience that can provide guidance on optimal antibiotic therapy [8]. Antibiotics used in prior published cases included imipenem, gentamicin, ciprofloxacin, vancomycin and ceftriaxone. A study from Japan characterized 13 isolates of *Gordonia bronchialis*, mostly from pulmonary samples obtained between 1998 and 2008, and these showed as susceptible to carbapenems and aminoglycosides with variable susceptibility to minocycline, vancomycin and third-generation cephalosporins, though with so few isolates tested this cannot be used as a definitive guide for empiric treatment [9]. Ceftaroline is a cephalosporin developed primarily to treat methicillin-resistant *Staphylococcus aureus* (MRSA) infections and it is approved by the Food and Drug Administration (FDA) for skin and soft tissue infections, with or without bacteraemia. In this case, in the context of growth of MRSE and a low ceftaroline MIC for both the MRSE and the *G. bronchialis*, and clinical failure of prolonged vancomycin therapy, we opted to treat the patient with ceftaroline. We used a higher dose than the one that is FDA approved for skin and soft tissue infections and pneumonia, but it was within the range of what we have used to treat MRSA osteomyelitis [10]. Given the good outcome in this case and the paucity of clinical data to guide treatment, ceftaroline may be considered an alternative agent to complement adequate surgical debridement.
